#  Exploring the experiences of residents in managing multiple roles at tertiary care hospitals of Punjab, Pakistan

**DOI:** 10.12669/pjms.41.6.11508

**Published:** 2025-06

**Authors:** Irum Naz, Kinza Aslam, Ahsan Sethi

**Affiliations:** 1 Irum Naz Lecturer, Department of Anatomy, Khawaja Muhammad Safdar Medical College, Sialkot, Pakistan.; 2 Kinza Aslam Associate Professor, Department of Medical Education, University College of Medicine and Dentistry, The University of Lahore, Lahore, Pakistan.; 3 Ahsan Sethi Associate Professor, Health Professions Education, Department of Public Health, College of Health Sciences, QU Health, Qatar University, Doha, Qatar

**Keywords:** Multiple roles, Postgraduate training, Residents, Tertiary care hospitals

## Abstract

**Objectives:**

No qualitative studies have explored the multiple roles undertaken by medical residents in Pakistan or the impact these roles have on them. This study explores the experiences of medical residents in managing their multiple roles at tertiary care hospitals in Punjab, Pakistan.

**Methods:**

An exploratory qualitative study was conducted from February 2023-2024. Using maximum variation purposive sampling, 12 residents across various specialties from four tertiary care hospitals of Punjab, Pakistan were interviewed after informed consent. The data were transcribed, and thematic analysis was performed.

**Results:**

The participants reported being involved in providing clinical services, teaching, management of patient records, logistics, and ward operations alongside their training. These multiple roles had a profound impact on their health, academics, research, and provision of services during the training. They also highlighted some challenges and enablers towards performing these roles and responsibilities, e.g., security issues, patient load, politics, and resources.

**Conclusion:**

Postgraduate residents perform multiple roles during their training, which impacts their health, academics, and patient safety. There is a need for professional development of the supervisors and residents to ensure that the training and development are not overshadowed by ancillary responsibilities. A structured training program with clear job descriptions, contracts, orientation, and continuous assessment may help improve the experiences of residents.

## INTRODUCTION

Postgraduate medical residents operate within intricate, unforeseeable adaptive frameworks fraught with sudden challenges in healthcare institutions.^1^ Due to the demanding nature of evening shifts and the long hours of practice, achieving this goal in the workplace poses a significant challenge.^2^ Residents are expected to fulfill multiple roles, including caregiver, communicator, researcher, educator, manager, and leader.^3^ Postgraduate residents are prone to encountering various pressures, such as time constraints and the challenge of balancing multiple responsibilities. These stressors can lead them to feel demoralized and disconnected from their own identity.^4^,^5^

Residents endure significant stress due to the demanding nature of their training.^6^ Additionally, they grapple with the challenges of providing services and medical care to the patients. Difficult aspects of doctor-patient roles involve delivering bad news, understanding beliefs, resolving conflicts, supporting behavior change, addressing mental health, and language barriers.^7^ Various studies have reported the potential negative impact of job-related stress and psychological tension among health professionals on patient management.^4^,^8^ The multiple roles the residents encounter affect their professional and emotional well-being, which is crucial for adequate healthcare delivery and patient safety.

There is limited literature on the impact of multiple roles related to service delivery on the learning and development of postgraduate residents. No qualitative studies have explored these multiple roles in-depth and their impact on postgraduate residents in Pakistan. The current study explores the various roles performed by postgraduate residents in tertiary care hospitals in Punjab, Pakistan. It also identifies the challenges and enablers towards performing these roles and their impact on personal and professional life.

## METHODS

An exploratory qualitative study was conducted from February 2023-2024. Participants included postgraduate residents (PGRs) with more than one year of training from four tertiary care hospitals across Punjab, Pakistan. All four hospitals were accredited by the College of Physicians and Surgeons Pakistan (CPSP) for postgraduate training in various specialties. Using purposive maximum variation, 12 medical residents (three from each hospital) were invited to participate in the study.

### Ethical Approval:

It was granted by the Ethics Review Board of the University of Lahore (Reg. No. 13/23/01, Dated: February 03, 2023).

The authors developed a semi-structured interview guide based on the literature review on medical residents’ roles, work-related stress, and postgraduate training challenges^1^-^5^. It was then validated by six health professionals with expertise in qualitative research. It was subsequently piloted with postgraduate residents (n=2) to ensure clarity and comprehensiveness. An informed consent was obtained by the first author, who also conducted all the interviews. Interviews were recorded on two devices as a precaution, and notes were also taken. Confidentiality and anonymity were strictly maintained due to the sensitive nature of the topic. All interviews were transcribed verbatim and a thematic analysis was conducted following Braun and Clark’s Six-phase framework. Analysis involved inductive coding through iterative cycles, organizing them into meaningful subthemes and themes. All the authors analyzed the data independently and agreed upon themes after deliberation. Reflexivity was practiced throughout to ensure credibility and trustworthiness.

## RESULTS

The participants included both genders, represented various specialties, and came from four tertiary care hospitals across Punjab, Pakistan (Table-I). The participants reported being involved in providing clinical services, teaching, management of patient records, logistics, and ward operations alongside their training. These roles and responsibilities take priority over their primary role as postgraduate residents, which is training and development. Some of these roles and responsibilities are not perceived as part of being a postgraduate resident (Table-II). Participants highlighted various challenges in performing their roles as residents (Table-III). They also outlined some enablers for managing the array of roles and responsibilities during their training (Table-IV). The participants outlined the impact of multiple roles and responsibilities on their health, academics, research, and provision of services during the training (Table-V).

**Table-I:** Participant Characteristics.

Characteristics		Frequency	Percentage (%)
Gender	Male	7	58.3
	Female	5	41.7
Training Year	Second	3	25
	Third	5	41.7
	Fourth	4	33.3
Department	General Medicine	5	41.6
	General Surgery	2	16.7
	Orthopedics	1	8.3
	Ophthalmology	2	16.7
	Gynecology	2	16.7

**Table-II:** Roles and Responsibilities of Residents.

Training and Development		“To present daily patient cases during ward rounds, give our monthly assessments, make and deliver presentations on various topics, participate in workshops, conduct research, and submit a thesis before the end of training”. (P8)
Clinical Services		“As a clinical trainee, I have to perform my duties in the wards, OPD, and OT for the care of patients, and make clinical judgments according to situations” (P1)
Teaching and Supervision		“I am supposed to obey all the orders given to me by seniors and consultants. I have to supervise my junior residents and house officers”. (P3) “…and teaching medical students during their ward rounds.” (P10)
Management	Clerical tasks	“We are also responsible for the audit of the patients and keeping records of the admissions, discharges, and mortality of the patients in the ward even though it doesn’t fall under our responsibilities”. (P6)
	Operational support	“We are also assigned the duties of managing the cleanliness of the wards and the roster is being made every week for that. We have to deal with paramedical staff and nurses as well who are not willing to perform their assigned duties” (P4)
	Logistical responsibilities	“We have to perform the roles that we are not supposed to do. Taking calls ourselves to other departments, calling consultants personally from other departments, and even arranging for blood and running to the blood center are the roles we perform in the wards and emergency”. (P5)
	Handling attendants	“We have to deal with the attendants of the patients as well due to poor management systems and limited or less space in the trauma center”. (P4)

**Table-III:** Challenges in performing roles.

Security issues	“We are many times threatened by aggressive attendants who are always ready to beat the doctors in the hospitals. No security provided to the doctors in the hospitals makes them vulnerable to harassment and threats by the attendants”. (P5)
Patient load	“There is an overburden of patients in tertiary care hospitals and a shortage of doctors who have to manage a number of patients”. (P3)
Poor management system	Our system to deal with Medicolegal cases is very poor in our hospitals which makes attendants aggressive leading to problems”. (P5)
Internal politics	“Departmental politics is usually a part of the OBG department which is a leading cause of stress and anxiety in the trainees”. (P7)
Lack of support from seniors and consultants	“I usually face conflicts due to the behavior of seniors. They are always ready to leg-pull the juniors. It creates a tense environment in the department which ruins my mental health.”. (P2)
Limited Resources	“More staff and doctors are needed in our clinical settings. Nobody can perform their best due to a lack of facilities, fewer resources, and more working hours”. (P9)
VIP Protocol disruptions	“Once during emergency duty due to a visit of some politicians, all ward boys were asked to clean the ward instead of helping patients and doctors. I being a senior duty doctor, objected to this act which led to conflicts between hospital DMS and me”. (P11)

**Table-IV:** Enablers towards performing roles.

Positive reinforcement from senior colleagues	“Some seniors are supportive and are always ready to help the juniors. They are the prime enablers and help us in our working environment”. (P2)
Support from family	“My family is a big supporting pillar for me who motivates and encourages me in my hard times”. (P9)

**Table-V:** Impact of juggling multiple roles.

Personal Impact	Work-life imbalance		“Balancing clinical duties with personal life is difficult sometimes”. (P9)
	Burn-out		“Physical, mental, and social well-being is affected due to overburden of work and departmental politics. When you are always occupied with work and have no time for yourself and your family, it causes anxiety, depression, and less interest in your work”. (P8)
Professional Impact	Academic	Limited study hours	“I find it quite difficult to find time for my studies due to my hectic work routine. My IMM exam got delayed because I was not able to prepare for my exam due to time management”. (P1)
		Delayed research completion	“Academic challenges like getting your synopsis done on time as well as preparing for the IMM and FCPS-2 examination are always there for the trainees. We are so overburdened with the tasks provided to us that we do not get time to focus on our academics”. (P3)
		Job dissatisfaction	“Sometimes, I feel that choosing training in Pakistan was not a good decision to be made as the stress is more and the pay scale is not up to the mark”. (P9)
	Services	Compromised patient care	“When we are already overburdened, it has an impact on the quality of the provision of clinical services to patients. We are busy with so many other tasks that patient care is ultimately affected”. (P5)

## DISCUSSION

The current study explores the roles and responsibilities, their enablers, challenges, and impact on postgraduate residents at tertiary care hospitals in Punjab. The participants highlighted their roles as mentors, alongside their clinical duties. Recent studies emphasize the importance of resident trainees as mentors and educators in instilling professional principles in colleagues and students.^9^ Some participants also disclosed that interacting with nurses and paramedical staff presents a challenging aspect of their role. This aligns with existing literature, which suggests that the interprofessional teams in healthcare have led to increased conflicts between physicians and nurses. These conflicts often stem from differing perspectives on collaboration and decision-making processes.^10^ This suggests a need for the development of collective competence through interprofessional learning and collaborations.

Some managerial roles and responsibilities are not perceived as part of being a postgraduate resident, which suggests a need for clear job descriptions, contracts, and orientation sessions at the induction of the postgraduate residents. Also, workplace-based assessment tools such as multisource feedback should be implemented to continuously review and provide feedback on the residents’ sense of responsibility, management skills, self-directed learning, teamwork, and patient care.^11^ There is also a need for structured training for the supervisors as well as the residents to ensure that other roles and responsibilities of the residents do not take priority over their primary role of training and development.^12^,^13^

The key enablers were positive reinforcement from senior colleagues and support from the families. The senior colleagues have a crucial role in assisting junior residents in navigating the demanding and fast-paced hospital environment. The stress associated with one’s job or occupation is mitigated by the support received from coworkers and family members.^8^,^14^ Motivation and passion in the field of study are also the key to successful retention and success during postgraduate training.^8^,^15^ The participants reported various challenges related to security issues, patient load, poor management system, internal politics, lack of support from seniors, limited resources, and VIP protocols. Some of these challenges have been commonly reported in the literature.^4^,^8^,^16^-^19^ The participants reported these challenges’ direct and indirect impact on their physical, mental, and social well-being.^17^ Burnout is often attributed to prolonged working hours, and physical and mental exhaustion emerge as common symptoms.^20^ Increased workload is associated with heightened strain, such as work-related stress and job burnout, which in turn impacts performance.^21^ The ineffective training coupled with insufficient support from seniors in clinical training has been identified as a contributing factor to a stressful experience.^22^

Additionally, irrelevant work demands can also adversely affect the overall well-being of health professionals.^23^ The work-life imbalance is also a significant contributor to work stress among healthcare professionals.^24^ Factors such as heightened job expectations, long working hours, shift work, and staff shortages have been identified as contributing to an imbalance between work and personal life.^25^ Reduction in duty hours, appropriate spacing in long shifts, more support by supervisors, and other perks for residents may help.^4^ All these challenges also impacted their exam preparation, research, and scholarly activities.

The literature also highlights a direct correlation between time management and academic performance. Effective time management fosters self-efficacy, thereby enhancing motivation and academic performance.^26^ Likewise, they felt job dissatisfaction, which consequently may have influenced their attitude towards their training and patient care.^19^ Patient safety is increasingly recognized as a major concern today, which is often compromised due to various preventable causes such as poor communication among health professionals and other systemic vulnerabilities in organizational procedures and management practices.^27^

This is the first in-depth qualitative exploration of postgraduate residents’ experiences of managing multiple roles at tertiary care hospitals of Punjab, Pakistan. Unlike prior research on stress, burnout and learning environment,^28^,^29^ the current study highlights the often-overlooked ancillary responsibilities in clinical settings and their personal and professional impact on residents.

The findings highlight the urgent need for clear role delineation and policies that prioritize education over service delivery in residency programs. Clinically, overburdening residents with non-core tasks may compromise patient safety due to fatigue and reduced focus. Educationally, the study advocates for reshaping training environments to align with global standards and support resident well-being.

### Limitations

The sample size of 12 participants, though suitable for comprehensive understanding in qualitative research, may limit representativeness and generalisability. Participants belonged to public-sector tertiary care hospitals in Punjab, which may not reflect experiences in private-sector, rural settings, and hospitals outside Punjab. Also, the authors are academics, which might have impacted our ability to fully grasp the nuanced challenges and conflicts embedded in clinical practice. However, this externality allowed us to identify patterns or issues that insiders might overlook. Moreover, reflexivity and analytical triangulation were employed to enhance credibility.

## CONCLUSION

Postgraduate residents perform multiple roles during their training, which impacts their health, academics, and patient safety. There is a need for professional development of the supervisors and residents to ensure that the training and development are not overshadowed by ancillary responsibilities. A structured training program with clear job descriptions, contracts, orientation, and continuous assessment may help improve the experiences of residents.

### Recommendations

Future studies should longitudinally study the experiences of residents, their career progression, and impact on professional identity over time.

### Author’s Contribution:

**IN and AS** conceptualized the study and designed the methodology.

**IN** collected the data.

All the authors were involved in the data analysis, interpretation, writing, and approval of the manuscript. They are also accountable for the integrity of the study.
